# Efficacy of acupoint-related therapies for nausea and vomiting in pregnancy: a Bayesian network meta-analysis

**DOI:** 10.3389/fmed.2025.1589950

**Published:** 2025-09-30

**Authors:** Hejing Liu, Cai Liao, Junyuan Deng, Yunhao Yang, Yan Yang, Xiao Guo, Chunshan Liu, Chenglin Tang

**Affiliations:** ^1^College of Traditional Chinese Medicine, Chongqing Medical University, Chongqing, China; ^2^College of Acupuncture and Tuina, Chongqing College of Traditional Chinese Medicine, Chongqing, China; ^3^Chongqing Academy of Chinese Materia Medica, Chongqing, China

**Keywords:** acupoint, pregnancy, nausea and vomiting, Bayesian, meta-analysis

## Abstract

**Objective:**

Bayesian network meta-analysis was used to compare the efficacy of different acupoint-related treatments for Nausea and Vomiting of Pregnancy (NVP).

**Methods:**

PubMed, Embase, Cochrane Library, CNKI, Wan Fang, and VIP databases were systematically searched from the time of library construction to February 20, 2025, to include randomized controlled trials (RCTs) comparing acupoint-related treatments for the treatment of nausea and vomiting in pregnancy. Literature screening, data extraction and risk of bias assessment were performed independently by two investigators, Bayesian network Meta-analysis was performed by R4.4.1 software.

**Result:**

A total of 38 studies containing 1,164 patients were included, this Bayesian network meta-analysis assessed the efficacy of various treatments for NVP across multiple outcomes. Results indicated that Acupoint Application (AA), Acupressure, Auriculotherapy Acupoint Application (ATAA), Ginger Moxibustion Acupoint Application (GMAA), and Moxibustion Acupoint Application (Mox_AA) were significantly more effective than Press Needle (PN) in improving PUQE scores. Thunder Fire Moxibustion (TFM) ranked highest in efficacy (89.1%), followed by GMAA (74.2%) and Acupressure (70.3%). Regarding overall efficacy, AA was less effective than AA_WA (OR = 0.22) and Acupuncture (OR = 0.44), but more effective than usual care (UT) (OR = 3.76), with AA_WA ranking highest (84.7%). In terms of NVP quality of life, TCM_acupuncture showed the greatest benefit (MD = 30.43), significantly outperforming AA (MD = −42.54), Mox_AA, and UT. Overall, TCM_acupuncture emerged as the most effective treatment for both symptom relief and quality of life improvement, followed by Mox_AA and ATAA, while UT was the least effective across all measures.

**Conclusion:**

Overall, the analyses showed that TFM may be the most effective in treating NVP, followed by GMAA and ear pressure therapy. Compared to PN, AA, auricular pressure therapy, ATAA and GMAA were more effective. AA was more effective than UT, but not as effective as AA_WA and acupuncture. In terms of quality-of-life improvement, TCM_acupuncture may be the most effective, followed by Mox_AA and ATAA, and UT was the least effective. Overall, acupuncture-based treatments, especially Thunder Fire Moxibustion and TCM acupuncture, performed better in the treatment of NVP.

## Introduction

Nausea and Vomiting in Pregnancy (NVP) is one of the most common symptoms during pregnancy, affecting approximately 50 to 80 per cent of pregnant women worldwide usually occurs early in pregnancy and is accompanied by symptoms such as nausea, vomiting, and loss of appetite, which in severe cases may affect the quality of daily life of the pregnant woman (1). Although NVP is usually considered a benign phenomenon, its clinical presentation and extent vary widely, with some pregnant women experiencing severe symptoms leading to severe vomiting of pregnancy, often accompanied by dehydration, electrolyte imbalance, and significant weight loss, which may endanger the health of the mother and baby and even require hospitalization (2). Therefore, effective management of NVP is of great significance in safeguarding the health of mothers and infants.

Currently, the treatment of NVP relies heavily on pharmacological interventions, and common medications include antihistamines, antiemetics, and, in some cases, steroids (3). Some drugs may have adverse effects on the fetus, antihistamines may cause fetal growth restriction and steroids may lead to fetal weight loss. In addition, prolonged reliance on drug therapy may lead to accumulation of drugs in the pregnant woman’s body, further increasing the risk of fetal exposure (4). Therefore, more and more pregnant women and clinicians are seeking non-pharmacological treatments, especially those based on traditional medicine. In recent years, acupoint-related therapies have gradually become a popular choice for relieving NVP symptoms (5). As a non-pharmacological treatment, meridian point therapy is believed to relieve symptoms such as nausea and vomiting by regulating the body’s qi, blood and internal organ functions (6). In addition, acupuncture is safe, especially for pregnant women, and can provide effective treatment without introducing medication side effects.

Although many studies (7, 8) have examined the therapeutic effects of different acupoint therapies on NVP, the results of the existing studies are still subject to a large degree of uncertainty due to the wide variety of therapies and the wide variation in study designs. On the one hand, some studies have shown that acupoint therapy has significant efficacy in reducing the symptoms of NVP, but others have failed to find significant differences (9, 10). On the other hand, the comparison and synthesis of treatment effects have become more difficult because different studies have used different treatment methods, treatment times, and evaluation indicators. Therefore, there is a lack of a systematic analysis that comprehensively assesses the relative efficacy of different meridian therapies (11). Network Meta-analysis, as an advanced statistical method capable of comparing multiple interventions simultaneously and synthesizing the efficacy of different treatments through indirect evidence, is an effective tool to address the above issues. Unlike traditional Meta-analysis, network Meta-analysis is not only able to directly compare the results of different studies of the same intervention, but also able to compare the relative efficacy between different treatments through indirect evidence. This study will provide a more scientific basis for clinical practice and help clinicians to choose the most effective and safest acupoint therapies for the treatment of NVP, thus providing better treatment options for pregnant women.

## Methods

The systemic review was supported by the online PROSPERO international prospective register of systemic reviews (12) of the National Institute for Health Research (CRD42025638660, https://www.crd.york.ac.uk/PROSPERO/view/CRD42025638660) and follow PRISMA-NMA guidelines.

## Literature retrieval

PubMed, Embase, Cochrane Library, CNKI, Wan Fang, and VIP databases were searched from database creation to February 20, 2025 search terms were acupuncture, Ear Acupuncture. The search terms were acupuncture, Ear Acupuncture, Auricular Acupuncture, Moxibustion, Nausea and vomiting, and the specific search terms are listed in Supplementary Table S1.

### Criteria for inclusion and exclusion

#### Inclusion criteria

The population included in this study was pregnant women diagnosed with NVP. Mild (episodic nausea and vomiting) and severe (severe vomiting of pregnancy) cases were included. At least one acupoint-related treatment (acupuncture, auricular pressure bean, acupoint patch, acupressure, interstitial ginger moxibustion, warm needle moxibustion, electroacupuncture) was compared in the study with a control treatment (the conventional care group received standard clinical management for nausea and vomiting during pregnancy, including lifestyle modifications and pharmacological treatments such as vitamin B6 or antihistamines. The placebo group received an inactive treatment designed to mimic active treatment but without therapeutic effects. Pharmacological comparators included commonly prescribed medications such as antihistamines, ondansetron). The primary outcomes were efficacy (cessation of nausea and vomiting, elimination of symptoms, and no recurrence after treatment is stopped), PUQE (Pregnancy-Unique Quantification of Emesis and Nausea) Score, and the secondary outcome was (NVPQOL) Nausea and Vomiting of Pregnancy Quality of Life Scale. The type of research included this time is a randomized controlled study (RCTs).

#### Exclusion criteria

Cohort studies, case–control studies, observational studies, where pregnant women have other serious concurrent medical conditions (gestational hypertension, diabetes), or where the study population consists of other non-pregnant cases of nausea and vomiting (gastroenteritis, post-chemotherapy vomiting).

### Data screening and data extraction

Two authors independently used endnote 21 for literature screening according to the inclusion and exclusion criteria, and if there was any controversy, it was discussed or a third person was sought for adjudication, and an excel sheet was used for data extraction, and the extracted data contained the first author, year of publication, sample size, mean age, Gestational age, interventions, and outcomes.

### Risk of bias

In this study, quality evaluation was assessed using the ROB (13). The ROB tool is used to assess the risk of bias in randomized controlled trials (RCTs) and consists of seven dimensions: random sequence generation, allocation concealment, blinding implementation, data completeness, selective reporting, other sources of bias, and study funding. The risk of bias for each aspect was categorized as high, medium or low. Specifically, studies were rated as low bias if the methodology was clearly described and compliant, high bias if the methodology was opaque or likely to lead to systematic error, and moderate bias if no specific bias could be identified. In addition, the assessment of each bias will be judged in combination with the quality of trial design, execution and reporting. The application of this tool helps to ensure the quality and reliability of the included studies, thus improving the credibility of the results of this study. All assessments will be done by two independent reviewers, and in case of disagreement, agreement will be reached through discussion.

### GRADE assessment

In this study, the quality of evidence was assessed using the GRADE system (14). The GRADE (Grading of Recommendations Assessment, Development and Evaluation) system classifies evidence into four grades: high, medium, low and very low quality. The rating process considers factors such as study design, risk of bias, directness, consistency, precision and reported bias. High-quality evidence usually comes from RCTs or strong observational studies with sound study design, low risk of bias and reliable conclusions. Moderate-quality evidence indicates that the study may have some bias or design deficiencies, but the conclusions are still highly credible. Low-quality evidence reflects that there are major problems with study design or execution, the results are less reliable, and the conclusions may be affected by bias or other factors. Very low-quality evidence usually comes from studies that are of extremely poor quality, and the conclusions are not credible and need to be supported by more research.

### Data analysis

We conducted a Bayesian network meta-analysis using R4.4.1 software (R Foundation for Statistical Computing) with *a priori* fuzzy random effects models for multiple sets of trials, model fitting was conducted using four independent Markov chains, each running for 50,000 iterations. The first 10,000 iterations of each chain were discarded as burn-in to ensure the chains had time to converge to the target distribution. The remaining 40,000 iterations were retained for posterior analysis. The combined estimates and probabilities of each treatment being the best were obtained by Markov chain Monte Carlo methods (15). Model convergence was assessed by trajectory plots and Brooks-Gelman-Rubin plots dichotomous classification results were expressed as the posterior odds ratio or and its 95% confidence intervals (CI). We calculated the percentage of area under the cumulative ranking curve (SUCRA) to estimate the probability of optimal intervention. Network diagrams were drawn using STATA 15.0 with a pass-through macro command loaded. For the network diagram, each circle corresponds to a drug and the edges represent existing comparisons. The size of the circles is proportional to the number of patients included. Cumulative probability plots were drawn using the ggplot2 package. To assess the consistency and heterogeneity across the studies, we conducted heterogeneity analysis by calculating the I^2^ statistic, which quantifies the proportion of variability in effect estimates due to heterogeneity rather than sampling error. Values of I^2^ greater than 50% indicate substantial heterogeneity, and sensitivity analyses were performed to explore potential sources of this variability, such as variations in study designs, sample sizes, and baseline characteristics.

## Result

### Literature search results

**Figure 1:**
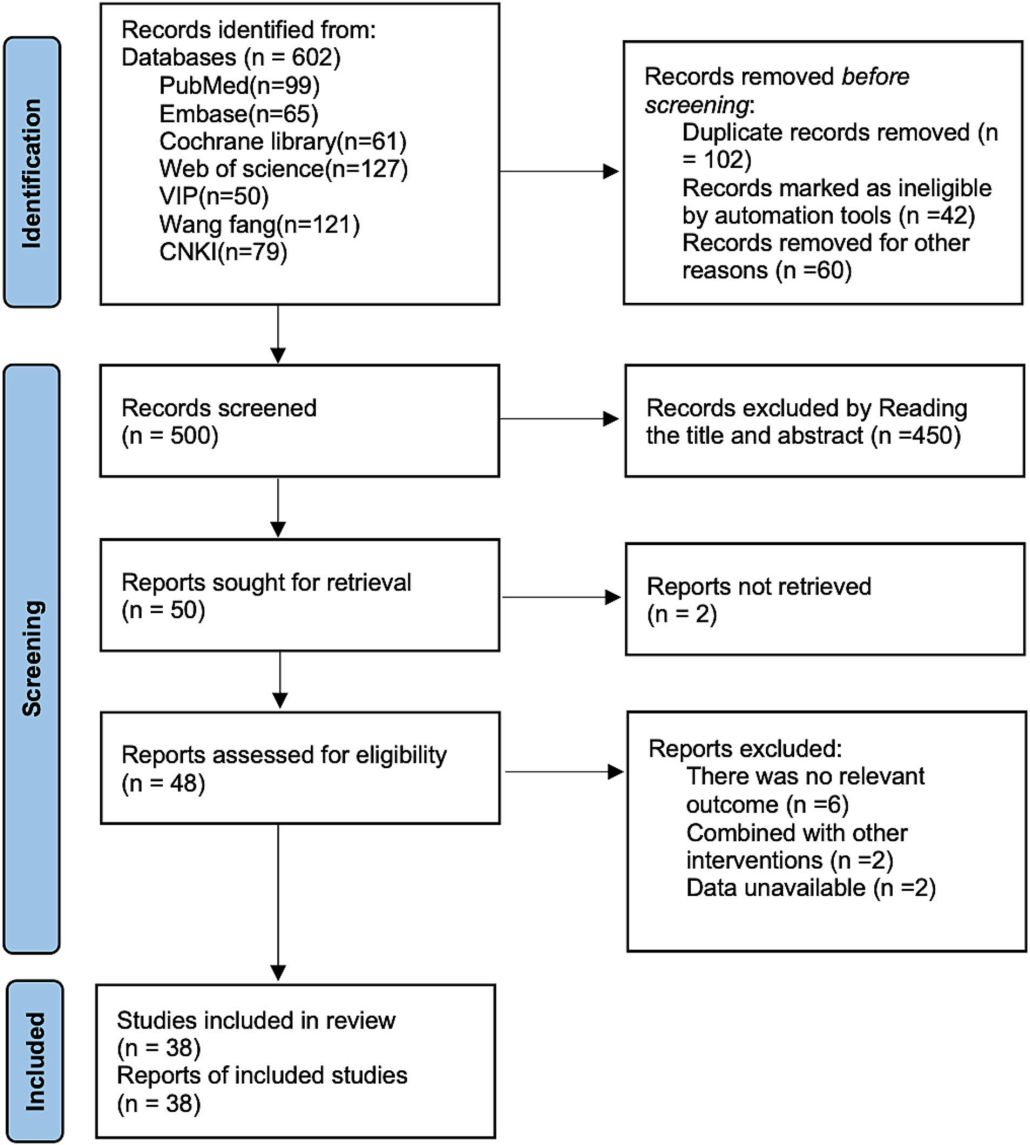
Literature search flow chart.

PubMed (*n* = 99), Embase (*n* = 65), Cochrane library (*n* = 61), Web of science (*n* = 127), VIP (*n* = 50), Wang fang (*n* = 121), and CNKI (*n* = 79) were systematically searched by removing 102 duplicates, by reading titles and abstract removal of 450, and removal of 12 by reading the full text, resulting in the inclusion of 38 randomized controlled studies (16–53). The specific flowchart for literature retrieval is shown in Figure 1.

### Basic characteristics of the included studies

A total of 38 studies containing 1,164 patients were conducted in which the interventions included, in total, 14 interventions Acupuncture, TCM_acupuncture, Moxibustion (Mox) and acupuncture_Mox, acupressure, auriculotherapy acupoint Application (ATAA), Acupoint application (AA), Scalp acupuncture acupoint application (SAAA), Press needle (PN), Thunder fire Moxibustion (TFM). Ginger moxibustion Acupoint Application (GMAA), Press needle (PN); Warm acupuncture (WA). The basic characterization table is shown in Table 1.

**Table 1:** Basic characteristics of literature.

Study	Year	Sample size	Mena age (year)	Gestational age (weeks)	Intervention	Outcomes
Adlan	2017	Acupressure:60 UT:60	Acupressure:29 UT:28.4	Acupressure:9.7 UT:9.2	Acupressure: P6	F1; F3
Nafiah	2022	Acupressure:45 UT:45	Acupressure:29.3 UT:30.8	Acupressure:10 UT:10.16	Acupressure: P6	F4; F5
Yılmaz	2023	Acupressure:37 UT:37	Acupressure:28.70 UT:28.84	Acupressure:9.32 UT:8.76	Acupressure: P6	F1;
Yxchang	2013	TCM_acupuncture:48 UT:48	TCM_acupuncture:22.6 UT:24.5	NR	TCM_acupuncture: Zhiyin	F6; F7
Xmchen	2022	ATAA:30 UT:30	ATAA:28.03 UT:27.88	ATAA 10.45 UT:10.39	ATAA	F1; F2; F8; F5
Cgying	2015	AA:30 UT:30	AA:25.09 UT:25.03	AA:10.08 UT:9.95	AA: Shenyue Point Danzhong	F6;
DNa	2022	TCM_acupuncture:30 UT:30	TCM_acupuncture:26.93 UT:26.77	TCM_acupuncture:7.87 UT:7.57	TCM_acupuncture: Dicang Sibai bucci	F2; F6
Yfan 2023	2023	SAAA:42 UT:42	SAAA:28.98 UT:28.29	SAAA:61.23d UT:65.59d	SAAA: Ziwu point	F1; F5
Ffang	2023	PN:35 UT:35	PN:29.35 UT:29.40	PN:8.05 UT:8.01	PN: Nei guan	F1; F6
Dxhong	2020	AA:38 UT:38	AA:27.05 UT:26.45	AA:8.5 UT:8.3	AA: Tiantu Zhongwan	F6
XfLi	2020	AA:62 UT:62	AA:28.41 UT:27.68	NR	AA: Zhongwan Tiantu Neiguan	F9; F6
YLi	2017	AA:34 UT:33	AA:23.34 UT:21.69	NR	AA: Liquorice Ginger processed Pinellia Achyranthes root	F6
YlLi	2020	AA:40 UT:40	AA:27.38 UT:27.17	AA:7.50 UT:7.55	AA Zhongwan	F6
HyLiao	2020	AP_WP:60 UT:60	AP_WP:25.24 UT:25.57	NR	AP_WP: Clove *Pinellia ternata*	F6; F8; F5
LLiu	2022	TCM_acupuncture:53 UT:53	TCM_acupuncture: UT:	TCM_acupuncture: UT:	TCM_acupuncture: Neiguan	F8; F5
SjLiu	2007	TCM_acupuncture:47 UT:47	TCM_acupuncture:25 UT:25	TCM_acupuncture: UT:	TCM_acupuncture: Neiguan zusanli	F6; F4
WLiu	2019	Mox:55 UT:55	Mox:34.48 UT:34.52	Mox:46.52d UT:46.48d	Mox: Zhongwan zusanli	F6
WxLiu	2011	Mox:30 acupuncture:30	Mox:32.1acupuncture:33.4	Mox: 46.2d acupuncture:44.8d	Mox: Zhongwan Neiguan zusanli	F6
ZnMao	2009	UT: 30 acupuncture:30	UT:28.87 acupuncture:28.23	UT:8.57 acupuncture:8.30	acupuncture: Zhongwan Zusanli	F6
JfNi	2022	AA:40 UT:40	AA:27.13 UT:27.17	AA:9.05 UT:9.01	AA: Zhongwan Neiguan	F6
YrNie	2022	GMAA:20 UT:20	GMAA:25.53 UT:25.73	GMAA:11.55 UT:11.73	GMAA: Neiguan zusanli	F1: F6: F8
YyWan	2023	TCM_PN:31 UT:30	TCM_PN:27.48 UT:26.83	TCM_PN:7.58 UT:7.47	TCM_PN: Neiguan zusanli	F1: F8
HWang	2023	AA:50 UT:50	AA:28.9 UT:28.7	AA:7.1 UT:7.4	AA: ZusanliZhongwan	F1: F2: F5: F8
TpWang	2024	Mox_AA:50 UT:50	Mox_AA:29.58 UT:28.14	Mox_AA:7.78 UT:8.14	Mox_AA: Zusanli Zhongwan Neiguan	F1: F2: F5: F6: F8
XWang	2015	Mox:40 UT:40	Mox:28.5 UT:27.2	Mox:58.3d UT:56.9d	Mox: Neiguan Zusanli	F6: F3
SfWu	2022	TFM:40 UT:40	TFM:27.4 UT:27.7	TFM:10.4 UT:9.7	TFM: Zhongwan Shangwan Zusanli	F1: F6
YhXu	2015	Acupuncture: 19 UT:17	Acupuncture: 26.7 UT:26.7	NR	Acupuncture: Zhongwan Neiguan Zusanli	F6
YXu	2015	Acupuncture _Mox:35 UT:35	Acupuncture _Mox:25 UT:25	Acupuncture _Mox: UT:	Acupuncture _Mox: Neiguan Zusanli	F6
Hpyang	2016	AA:38 UT:38	AA:31.3 UT:30.7	AA:10 UT:10	AA: Zhongwan Neiguan	F6: F9
HlYang	2021	AA_WA:44 UT:44	AA_WA:32.1 UT:29.1	AA_WA:9.25 UT:9.14	AA_WA: Neiguan	F6: F8
WhYe	2020	AA:62 UT:62	AA:21.5 UT:22.2	AA:12 UT:12	AA: neiguan	F1: F2: F5: F6: F8
CpZhang	2015	AA:38 UT:38	AA:26.5 UT:27.6	NR	AA: Zhongwan ShangwanZusanli	F6
HhZhang	2005	UT: 50 Acupuncture:50	UT: 31 acupuncture:26	UT: 20 acupuncture:17	acupuncture: Zhongwan Neiguan Zusanli	F6
HwZhang	2019	PN:40 UT:40	PN:28.2 UT:27.9	PN:7.5 UT:8	PN: Zhongwan Neiguan	F6
SzZhao	2024	PN:30 UT:30	PN:30.35 UT:30.64	PN:9.28 UT:9.31	PN: Neiguan	F1: F2: F5: F6: F8
LZhou	2024	Acupuncture:44 UT:44	Acupuncture:27.86 UT:28.48	Acupuncture:13.34 UT:13.28	Acupuncture: Neiguan Zusanli Zhongwan	F6

AA, Acupoint Application; PN, Press needle; WA, Warm acupuncture; Mox, Moxibustion; TCM, Traditional Chinese medicine; TFM, Thunder fire Moxibustion; GMAA, Ginger moxibustion Acupoint Application; ATAA, Auriculotherapy Acupoint Application; SAAA, Scalp Acupuncture Acupoint Application; UT, Usual care; F1, PUQE(Pregnancy-Unique Quantification of Emesis and Nausea) Score; F2, Hospital stay; F3, Satisfied; F4, Urine ketone levels; F5, Duration to achieve urine ketone clearance; F6, curative effect; F7, recurrence; F8, NVPQOL, Nausea and Vomiting of Pregnancy Quality of Life Scale; F9, Urine ketone levels; NR, Not reported.

### Risk of bias result

The current study used Revman 5.4 software to plot the risk of bias results, the results (Supplementary Figures S1, S2) suggested that nine studies (17, 27, 28, 30, 33, 35, 43, 45, 53) did not mention the randomization method shown to have been used and were therefore evaluated as unclear, and four articles (22, 26, 34, 40) accounted for the blinding method used and were evaluated as low risk. The summary table of risk of bias can be found in Supplementary Table S2. The grade ratings for this study are shown in Supplementary Table S3.

### Network meta-analysis result

#### Results of consistency modeling

The current study used a random-effects model to compare the difference in Deviance Information Criterion (DIC) between consistent and inconsistent modeling, and the absolute value of the difference in DIC was <5, The results (Supplementary Table S4) indicate that efficacy, PUQE Score, and NVPQOL have consistency.

#### PUQE (pregnancy-unique quantification of Emesis and nausea) score

**Figure 2:**
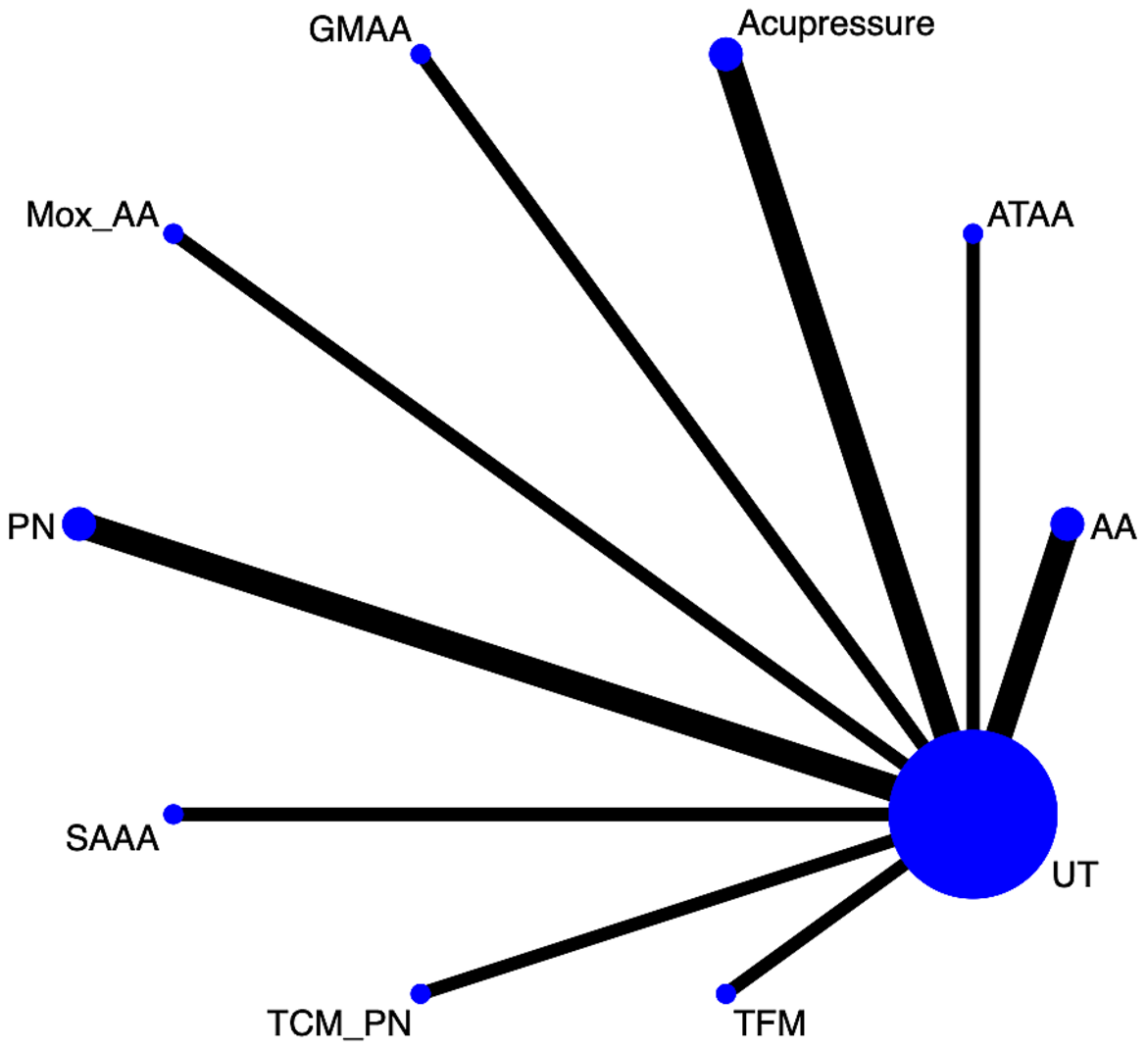
PUQE network diagram.

**Figure 3:**
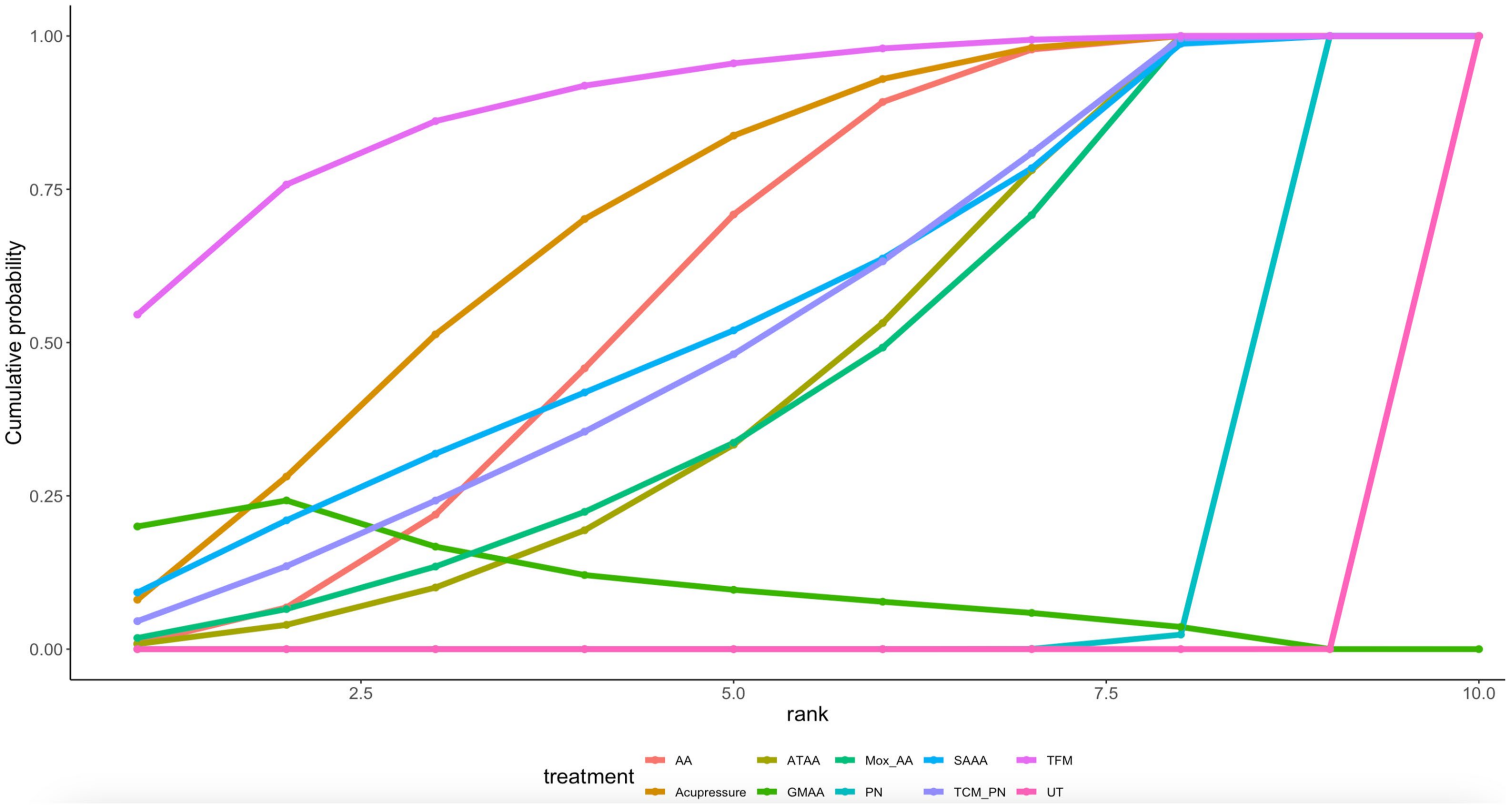
PUQE cumulative probability ranking.

Twelve article mentions PUQE scores, suggesting a direct comparison of UT with TFM, TCM_PN, SAAA, PN, Mox_AA, GMAA, Acupressure, ATAA, AA through a network diagram (Figure 2). Using the league table (Supplementary Table S5), it can be concluded that AA [MD = −1.24, 95%CI (−1.67, −0.81)], Acupressure [MD = −1.39, 95%CI (−1.97, −0.81)], ATAA [MD = −1.02, 95%CI (−1.65, −0.39)], and GMAA[MD = −1.48, 95%CI (−2.29, −0.68)], MOX_AA[MD = −0.99, 95%CI (−1.77, −0.21)] were better than PN. Ranking by area under the cumulative probability curve (Figure 3 and Table 2) yielded TFM (89.1%) > GMAA (74.2%) > Acupressure (70.3%) > UT (0.09%).

**Table 2:** SUCRA ranks results.

Treatment	PUQE score (%)	Efficacy (%)	NVPQOL (%)
AA_WA	NR	84.7	35.7
AA	59.3	34.2	60.9
Acupressure	70.3	NR	NR
ATAA	44.3	NR	64.5
GMAA	74.2	70.6	60.5
Mox_AA	44.1	52.5	82.9
PN	11.3	37.2	21.8
SAAA	55.2	NR	NR
TCM_PN	52.2	NR	NR
TFM	89.1	52.6	NR
UT	0.07	16.9	0.81
Acupuncture	NR	69.8	NR
Acupuncture_mox	NR	35.4	NR
TCM_acupuncture	NR	68.4	99.9
TCM_ PN	NR	NR	22.9
Mox	NR	43.0	NR

AA, Acupoint Application; PN, Press needle; WA, Warm acupuncture; Mox, Moxibustion; TCM, Traditional Chinese medicine; TFM, Thunder fire Moxibustion; GMAA, Ginger moxibustion Acupoint Application; ATAA, Auriculotherapy Acupoint Application; SAAA, Scalp Acupuncture Acupoint Application; UT, Usual care.

#### Efficacy

**Figure 4:**
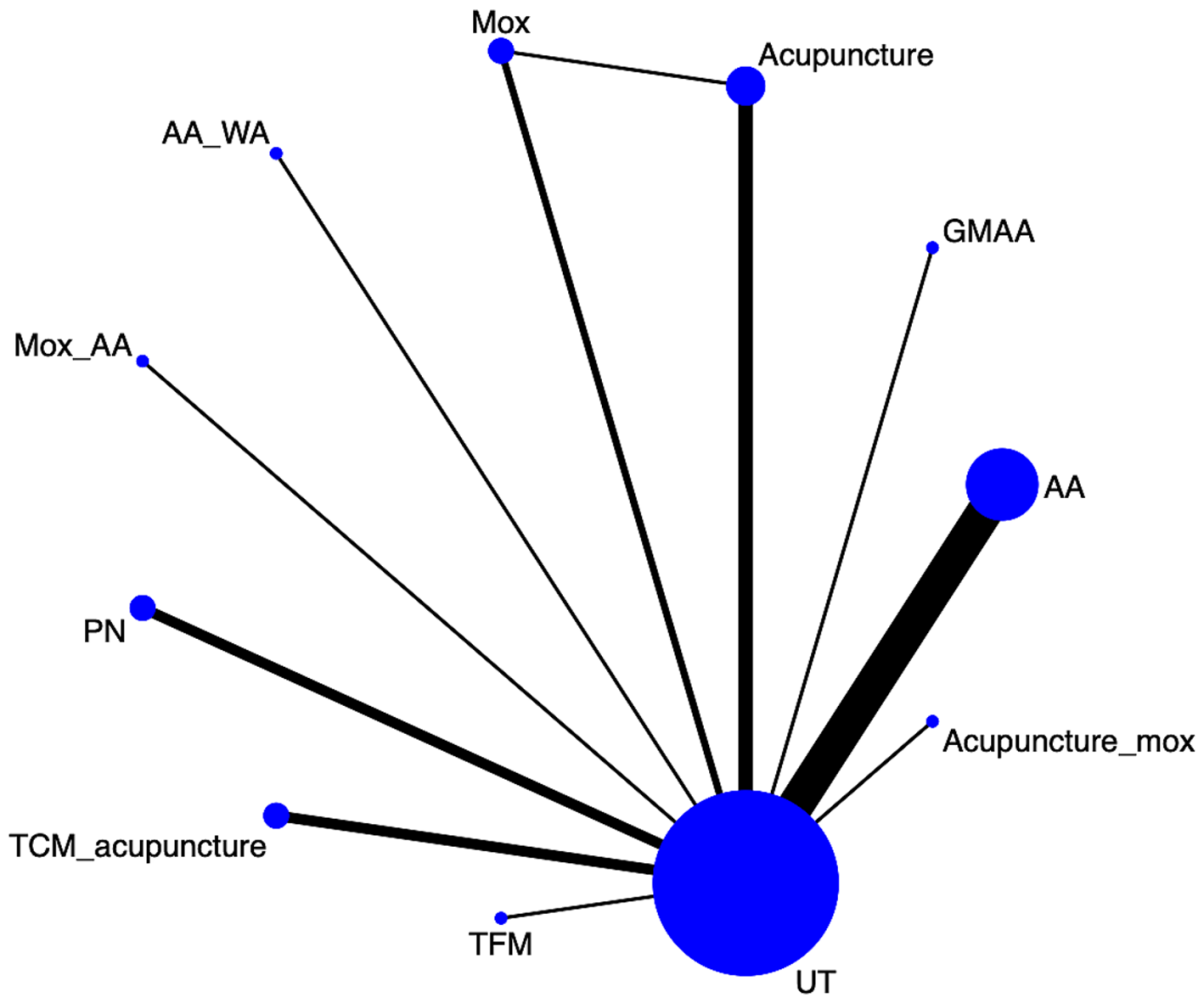
Efficacy network diagram.

**Figure 5:**
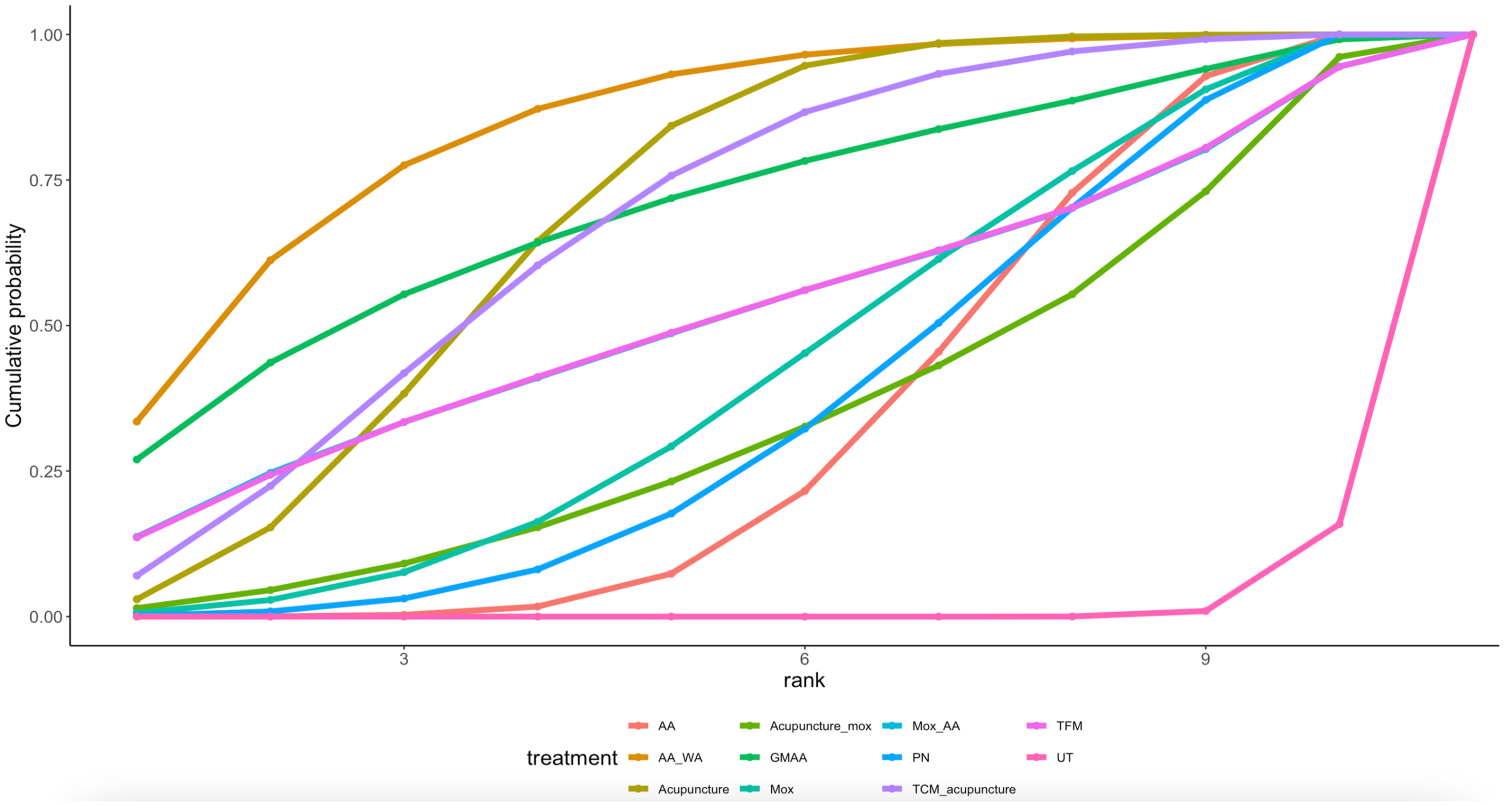
Efficacy cumulative probability ranking.

Twenty eight article mentions efficacy, suggesting a direct comparison of UT with, TFM; TCM_acupuncture; PN; Mox_AA; AA_WA; Mox; acupuncture; GMAA; AA; Acupuncture_mox through a network diagram (Figure 4). Using the league table (Supplementary Table S6), it can be concluded that AA was worse than AA_WA [OR = 0.22, 95%CI (0.03, 0.93)] and Acupuncture [OR = 0.44, 95%CI (0.19, 0.97)], but better than UT [OR = 3.76, 95%CI (2.45, 5.95)]. Ranking by area under the cumulative probability curve (Figure 5 and Table 2) yielded AA_WA (84.7%) > Acupuncture (69.8%) > TCM_acupuncture (68.4%) > UT (16.9%).

#### Nausea and vomiting of pregnancy quality of life

**Figure 6:**
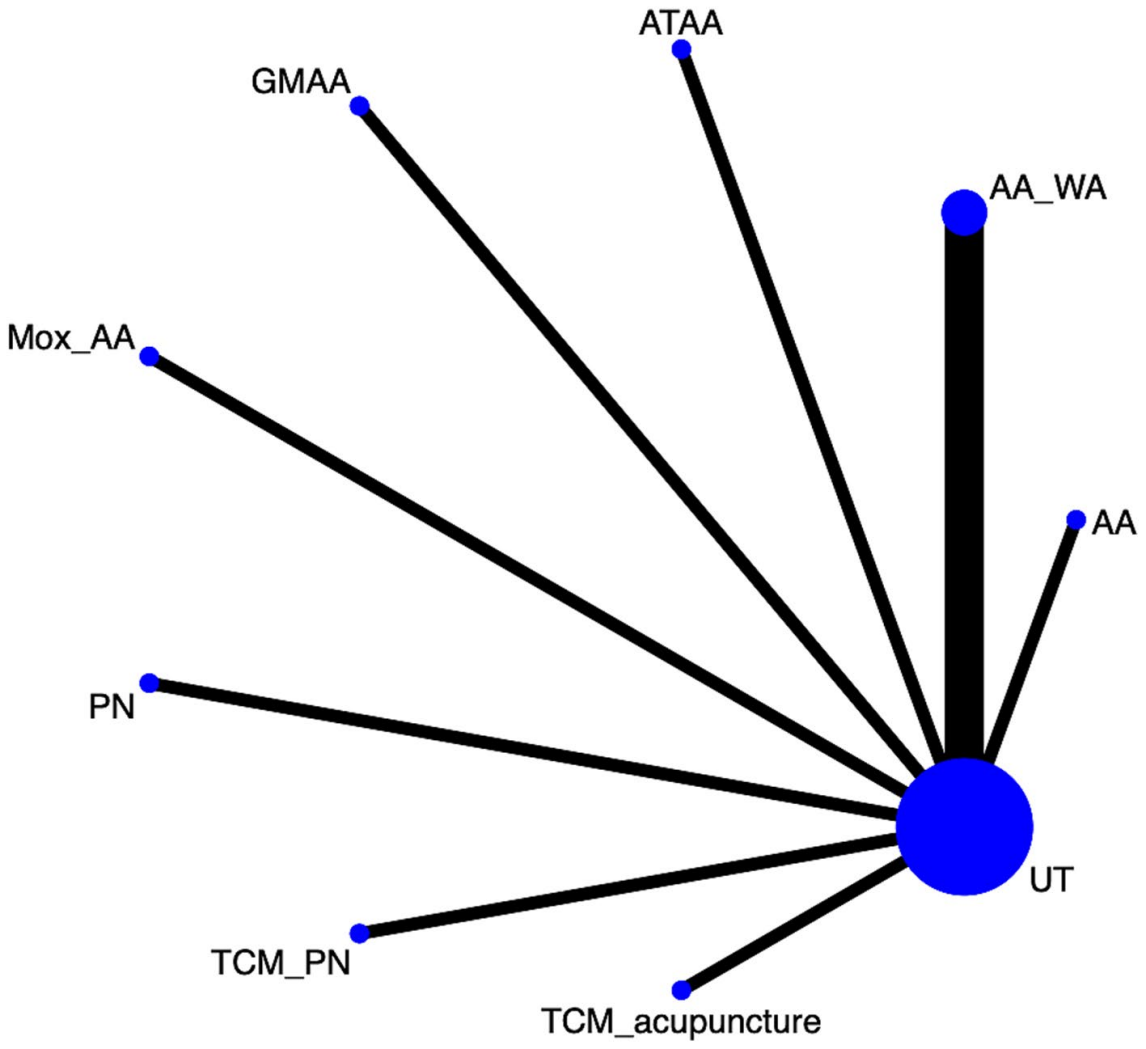
Nausea and vomiting of pregnancy quality of life network diagram.

**Figure 7:**
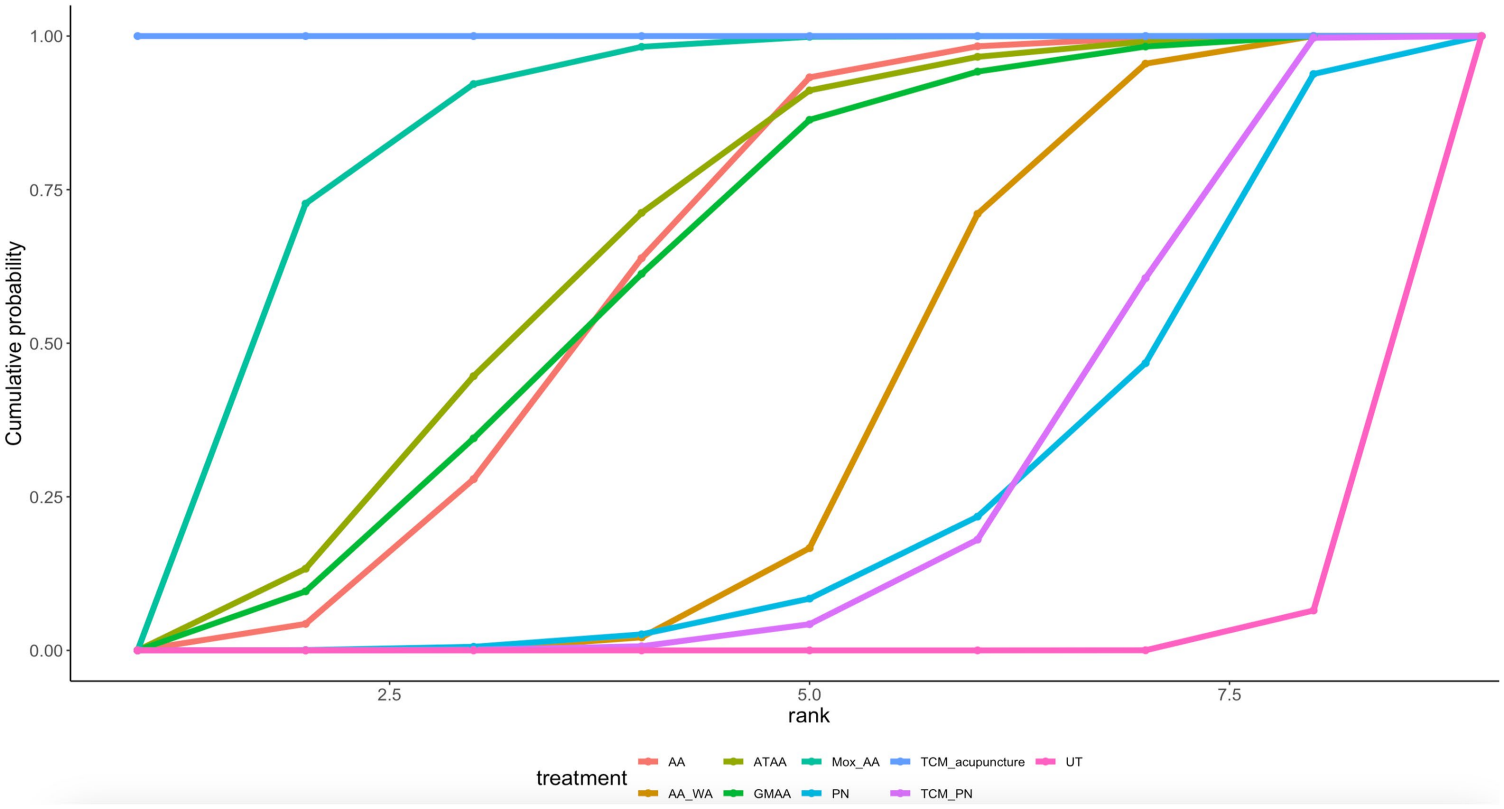
Nausea and vomiting of pregnancy quality of life cumulative probability ranking.

Ten article mentions NVPQOL, suggesting a direct comparison of UT with TCM_acupuncture, TCM_PN, PN, Mox_AA, GMAA, ATAA, AA_WA, AA through a network diagram (Figure 6). Using the league table (Supplementary Table S5, it can be concluded that AA was less effective than TCM_acupuncture [MD = 30.43, 95% CI (24.2, 36.65)]. TCM_acupuncture was more effective than TCM_PN [MD = −36.92, 95% CI (−42.67, −31.14)] and UT [MD = −42.54 (−46.7, − 38.38)]. Ranking by area under the cumulative probability curve (Figure 7 and Table 2) yielded TCM_acupuncture (99.9%) > Mox_AA (82.9%) > ATAA (64.5%) > UT (0.81%).

#### Publication bias

The current study used funnel plots and Egger test to detect publication bias, and the results (Supplementary Figures S3, S5) were asymmetric on both sides of the funnel plots and Egger’s results were efficacy(*p* = 0.03), PUQE Score(*p* = 0.001), and NVPQOL(*p* = 0.01), so publication bias can be assumed to exist.

## Discussion

To the best of our knowledge, this is the first time that a network meta-analysis has been used to explore different acupoint-related therapies, and our findings suggest that for PUQE scores: TFM (89.1%) > GMAA (74.2%) > Acupressure (70.3%) > UT (0.09%), efficacy: AA_WA (84.7%) > Acupuncture (69.8%) > TCM_acupuncture (68.4%) > UT (16.9%), NVPQOL: TCM_acupuncture (99.9%) > Mox_AA (82.9%) > ATAA (64.5%) > UT (0.81%).

In the Bayesian network Meta-analysis of this study, we analyzed the efficacy of different acupoint-related therapies on NVP in 12 studies. Comparison of PUQE scores showed that therapies such as AA, Acupressure, ATAA, GMAA, and Mox_AA had significant efficacy advantages over PN, and both therapies, GMAA and Acupressure in particular showed efficacy differences, but these results should be considered with the understanding that the quality of the evidence is moderate, and further studies with more robust designs are needed to confirm these findings. In addition, TFM showed a relatively high efficacy ranking (89.1%), suggesting its potential to reduce NVP symptoms. However, the results should be interpreted with caution due to the overall low-to-moderate quality of the available evidence (GRADE). Acupoint-related therapies are effective in relieving nausea and vomiting during pregnancy, and their efficacy may involve multiple biological mechanisms. The anti-emetic effects of therapies such as GMAA, which contains active ingredients like curcumin, have been explored in previous studies. These mechanisms may involve regulation of gastrointestinal motility and reduction of inflammation. In addition, ginger has anti-inflammatory effects (54), which may further relieve nausea by reducing inflammatory reactions in the gastrointestinal tract. Acupressure works by applying physical pressure to specific acupoints, such as Nei Guan acupoints, which promotes blood circulation, relieves gastrointestinal discomforts, and alleviates the feeling of nausea (49). TFM, as a special kind of moxibustion therapy, regulates the flow of qi and blood, and improves the function of the spleen and the stomach, through applying flames directly to the acupoints and stimulating the meridians through heat (55). Thunder Fire Moxibustion not only promotes blood circulation through the warming effect but may also alleviate the symptoms of NVP by activating the autonomic nervous system and enhancing gastrointestinal function (56). TFM, as a more intense form of moxibustion therapy, is effective in that it acts directly on the body through the flames, but the operation process requires special techniques and precautions. For pregnant women, as Thunder Fire Moxibustion involves direct heat stimulation, caution is needed to avoid burns during treatment. Compared to other treatments, TFM may require the operation of a professional therapist and is not suitable for all pregnant women, especially for those who are temperature sensitive or have skin allergies (57). From a clinical point of view, based on the results of this study, physicians may consider acupoint-related therapies more often in their treatment protocols, especially in the early stages of NVP. Compared with medication, these therapies are not only effective in relieving symptoms, but also offer better safety and fewer side effects, higher compliance among pregnant women, and can be implemented at home, reducing the burden of hospital visits (58).

For efficacy, AA_WA showed superior efficacy, which was significantly better than AA (auricular pressure bean combined with other therapies), while Acupuncture (acupuncture) also showed better efficacy. Compared to UT, AA (auricular pressure bean combined with other therapies) was significantly better in terms of efficacy. By ranking the area under the cumulative probability curve, we found that AA_WA had the highest ranking (84.7%), which indicated that it had the most significant efficacy in this analysis. By combining warm needling and auricular acupoint stimulation, AA_WA was able to effectively regulate the endocrine and autonomic functions. The advantage of this treatment is that its warming effect can directly affect blood circulation and metabolism, significantly accelerating the relief of symptoms, especially in improving central nervous system regulation and endocrine function (59). Acupuncture (69.8%) was the next most popular form of treatment, showing the significant role of traditional acupuncture in relieving symptoms. Acupuncture treatment not only regulates the autonomic nervous system, but also has anti-inflammatory and analgesic effects, which play an important role in relieving symptoms and promoting recovery (60). Doctors in clinical practice can make greater use of treatments with better efficacy, such as AA_WA and Acupuncture, which not only provide significant symptomatic relief, but also offer safer and more acceptable treatment options for patients, especially in the field of TCM treatment. Due to the non-pharmacological nature of Auricular Warming Needling and Acupuncture therapies, they have the potential for wide application in some patients who are unable or unwilling to use medications. It is important to note that most studies included in this analysis were conducted in China, which may limit the generalizability of the findings to other populations. Cultural factors, healthcare infrastructure, and treatment preferences in China may differ significantly from those in other countries. Therefore, while acupoint-related therapies have shown promise in the Chinese context, their effectiveness in diverse populations warrants further exploration. The quality of the evidence in this network meta-analysis was assessed using the GRADE framework, which considers factors such as study limitations, inconsistency of results, and imprecision. The overall evidence quality for the therapies assessed was considered low to moderate, which highlights the need for further high-quality randomized controlled trials to better assess the efficacy and safety of acupoint-related therapies for NV.

This suggests that TCM_acupuncture has a significant ameliorative effect on improving the quality of life in patients with nausea and vomiting during pregnancy. Through the area ranking under the cumulative probability curve, we found that TCM_acupuncture performed most prominently in improving the quality of life and ranked first (99.9%), which showed its significant therapeutic effect, while Chinese herbal medicine further improves the therapeutic effect by harmonizing the internal organs and dredging the meridians. This combination therapy has significant advantages in improving NVP and its quality of life. Closely followed by Mox_AA (82.9%), the therapeutic mechanism of moxibustion combined with auricular pressure beans mainly relieves nausea and vomiting by regulating the meridians, qi and blood, and the functions of the internal organs (61). TCM_acupuncture has a significant advantage in improving the quality of life of patients with nausea and vomiting during pregnancy, and therefore this method should be prioritized in clinical treatment, especially for those who wish to avoid the side effects of medications. This study exhibits significant heterogeneity, potentially stemming from variations in acupuncture techniques, treatment duration, and whether participants received baseline therapy—all of which represent potential sources of heterogeneity.

Although this study provides valuable evidence for the treatment of nausea and vomiting in pregnancy, some limitations remain:

1. Heterogeneity of included studies

Several different studies were included in this analysis, which involved varying treatment types, interventions, and patient characteristics. The differences in treatment protocols, sample sizes, and disease severity across the studies may contribute to heterogeneity in the results. This variability may affect the generalizability and robustness of the conclusions drawn from the analysis. While Bayesian network meta-analysis is a powerful tool for synthesizing results from multiple studies, caution should be exercised when interpreting these findings due to the potential for bias and variability across studies.

2. Variation in patient characteristics

The included studies varied widely in terms of patient age, disease duration, and symptom severity. For example, some studies targeted patients with mild nausea and vomiting of pregnancy, while others focused on those with more severe symptoms. These differences may have contributed to inconsistent efficacy assessments across studies. To enhance the generalizability and accuracy of future analyses, it would be beneficial for future studies to more precisely define patient characteristics and assess their impact on treatment outcomes.

3. Short-term focus and lack of long-term outcomes

This study primarily assessed short-term efficacy, with little consideration of long-term maternal and fetal outcomes (maternal safety, fetal outcomes, recurrence, cost-effectiveness). The management of nausea and vomiting in pregnancy should not only aim for immediate symptom relief but also consider long-term health effects for both the mother and the fetus. Unfortunately, most of the included studies did not evaluate long-term outcomes such as maternal health, fetal development, and quality of life over time. Future research should focus on assessing the long-term effects of these interventions, especially in terms of maternal and fetal health, to better understand the broader impact of treatment.

4. Evidence quality and generalizability

Most of the included studies were sourced from Chinese literature, which may limit the generalizability of the findings to broader populations. Additionally, many of the included randomized controlled trials (RCTs) had small sample sizes, which could undermine the reliability of the results. The overall quality of the evidence was rated as low to moderate according to the GRADE approach, with specific outcomes such as PUQE rated as low, efficacy as moderate, and quality of life as low. These factors should be considered when interpreting the conclusions of this analysis. In the future we will need more larger, multicenter, well-blinded RCTs outside China to prove our conclusion.

## Conclusion

This study compared the efficacy of various treatments in NVP by Bayesian network Meta-analysis. The results showed that TCM_acupuncture may be most significantly in relieving nausea and vomiting symptoms and improving the quality of life of pregnant women and ranked the highest. mox_AA and ATAA, AA_WA may also have better efficacy than UT. UT was less effective in improving symptoms and quality of life and ranked the lowest. Various TCM treatments are more suitable for pregnant women due to their low side effects and are better accepted by pregnant women. Overall, Acupoint-related therapies, such as TFM, GMAA, and Acupuncture, show potential in alleviating nausea and vomiting during pregnancy. However, given the moderate quality of evidence, these therapies should be considered adjunctive and used in conjunction with other standard treatments until higher-quality studies confirm their efficacy.

## Data Availability

The original contributions presented in the study are included in the article/Supplementary material, further inquiries can be directed to the corresponding authors.
