# Supplementary material for: Efficacy of acupoint-related therapies for nausea and vomiting in pregnancy: a Bayesian network meta-analysis
Source: Front Med (Lausanne). 2025 Sep 30;12:1589950. doi: 10.3389/fmed.2025.1589950 (PMC12518312; doi:10.3389/fmed.2025.1589950)
Supplement: Supplementary file 1 [file Data_Sheet_1.DOCX]

Table S1: specific search

| PubMed | ((((((((((("Acupuncture"[Mesh]) OR ((Acupuncture[MeSH Terms]) OR (Pharmacopuncture[MeSH Terms]))) OR ("Acupuncture Therapy"[Mesh])) OR (((((((((((Acupuncture Therapy[MeSH Terms]) OR (Acupuncture Treatment[Title/Abstract])) OR (Acupuncture Treatments[Title/Abstract])) OR (Treatment, Acupuncture[Title/Abstract])) OR (Therapy, Acupuncture[Title/Abstract])) OR (Pharmacoacupuncture Treatment[Title/Abstract])) OR (Treatment, Pharmacoacupuncture[Title/Abstract])) OR (Pharmacoacupuncture Therapy[Title/Abstract])) OR (Therapy, Pharmacoacupuncture[Title/Abstract])) OR (Acupotomy[Title/Abstract])) OR (Acupotomies[Title/Abstract]))) OR ("Acupuncture, Ear"[Mesh])) OR ((((((((Acupuncture, Ear[MeSH Terms]) OR (Acupunctures, Ear[Title/Abstract])) OR (Ear Acupunctures[Title/Abstract])) OR (Acupuncture, Auricular[Title/Abstract])) OR (Acupunctures, Auricular[Title/Abstract])) OR (Auricular Acupunctures[Title/Abstract])) OR (Auricular Acupuncture[Title/Abstract])) OR (Ear Acupuncture[Title/Abstract]))) OR ("Moxibustion"[Mesh])) OR ((Moxibustion[MeSH Terms]) OR (Moxabustion[Title/Abstract]))) OR ("Electroacupuncture"[Mesh])) OR ((((Electroacupuncture[MeSH Terms]) OR (Warm acupuncture[Title/Abstract])) OR (Dry needle[Title/Abstract])) OR (Fire needle[Title/Abstract]))) AND ((((("Nausea and vomiting"[MeSH Terms]) OR (Nausea[Title/Abstract])) OR (Vomiting[Title/Abstract])) OR (Emesis[Title/Abstract])) OR (Emeses[Title/Abstract]))) AND (("Pregnancy"[Mesh]) OR (((Pregnancy[MeSH Terms]) OR (Pregnancies[Title/Abstract])) OR (Gestation[Title/Abstract]))) |
| --- | --- |
| CNKI | 主题：(针灸 OR 温针灸OR 电针 OR 火针 OR 耳针 OR 针 OR 灸) and 主题：(恶心 OR 呕吐) |

Table S2 Summary table of risk of bias

| Study | Year | A | B | C | D | E | F | G |
| --- | --- | --- | --- | --- | --- | --- | --- | --- |
| Adlan | 2017 | Low risk | Unclear risk | Unclear risk | Unclear risk | Low risk | Low risk | Unclear risk |
| Nafiah | 2022 | Low risk | Unclear risk | Unclear risk | Unclear risk | Low risk | Low risk | Unclear risk |
| Yılmaz | 2023 | Low risk | Unclear risk | Unclear risk | Unclear risk | Low risk | Low risk | Unclear risk |
| Yxchang | 2013 | Low risk | Unclear risk | Unclear risk | Unclear risk | Low risk | Low risk | Unclear risk |
| Xmchen | 2022 | Low risk | Unclear risk | Unclear risk | Unclear risk | Low risk | Low risk | Unclear risk |
| Cgying | 2015 | Low risk | Unclear risk | Unclear risk | Unclear risk | Low risk | Low risk | Unclear risk |
| DNa | 2022 | Unclear risk | Unclear risk | Unclear risk | Unclear risk | Unclear risk | Unclear risk | Unclear risk |
| Yfan 2023 | 2023 | Low risk | Unclear risk | Unclear risk | Unclear risk | Low risk | Low risk | Unclear risk |
| Ffang | 2023 | Unclear risk | Unclear risk | Unclear risk | Unclear risk | Low risk | Low risk | Unclear risk |
| Dxhong | 2020 | Unclear risk | Low risk | Unclear risk | Unclear risk | Low risk | Low risk | Unclear risk |
| XfLi | 2020 | Low risk | Low risk | Unclear risk | Unclear risk | Low risk | Low risk | Unclear risk |
| YLi | 2017 | Low risk | Unclear risk | Unclear risk | Unclear risk | Low risk | Low risk | Unclear risk |
| YlLi | 2020 | Low risk | Low risk | Unclear risk | Unclear risk | Low risk | Low risk | Unclear risk |
| HyLiao | 2020 | Low risk | Low risk | Unclear risk | Unclear risk | Low risk | Low risk | Unclear risk |
| LLiu | 2022 | Low risk | Low risk | Unclear risk | Unclear risk | Low risk | Low risk | Unclear risk |
| SjLiu | 2007 | Low risk | Low risk | Unclear risk | Unclear risk | Low risk | Low risk | Unclear risk |
| WLiu | 2019 | Low risk | Low risk | Unclear risk | Unclear risk | Low risk | Low risk | Unclear risk |
| WxLiu | 2011 | Low risk | Low risk | Unclear risk | Unclear risk | Low risk | Low risk | Unclear risk |
| ZnMao | 2009 | Unclear risk | Low risk | Unclear risk | Unclear risk | Low risk | Low risk | Unclear risk |
| JfNi | 2022 | Low risk | Low risk | Unclear risk | Unclear risk | Low risk | Low risk | Unclear risk |
| YrNie | 2022 | Low risk | Low risk | Unclear risk | Unclear risk | Low risk | Low risk | Unclear risk |
| YyWan | 2023 | Low risk | Low risk | Unclear risk | Unclear risk | Low risk | Low risk | Unclear risk |
| HWang | 2023 | Low risk | Low risk | Unclear risk | Unclear risk | Low risk | Low risk | Unclear risk |
| TpWang | 2024 | Unclear risk | Low risk | Unclear risk | Unclear risk | Low risk | Low risk | Unclear risk |
| XWang | 2015 | Low risk | Low risk | Unclear risk | Unclear risk | Low risk | Low risk | Unclear risk |
| SfWu | 2022 |  |  |  |  |  |  |  |
| YhXu | 2015 | Unclear risk | Low risk | Unclear risk | Unclear risk | Low risk | Low risk | Unclear risk |
| YXu | 2015 | Low risk | Low risk | Unclear risk | Unclear risk | Low risk | Low risk | Unclear risk |
| Hpyang | 2016 | Low risk | Unclear risk | Unclear risk | Low risk | Low risk | Low risk | Unclear risk |
| HlYang | 2021 | Low risk | Unclear risk | Low risk | Unclear risk | Low risk | Low risk | Unclear risk |
| WhYe | 2020 | Low risk | Low risk | Unclear risk | Unclear risk | Low risk | Low risk | Unclear risk |
| CpZhang | 2015 | Unclear risk | Low risk | Unclear risk | Unclear risk | Low risk | Low risk | Unclear risk |
| HhZhang | 2005 | Low risk | Unclear risk | Unclear risk | Unclear risk | Unclear risk | Unclear risk | Unclear risk |
| HwZhang | 2019 | Unclear risk | Low risk | Unclear risk | Unclear risk | Low risk | Low risk | Unclear risk |
| SzZhao | 2024 | Unclear risk | Low risk | Unclear risk | Unclear risk | Low risk | Low risk | Unclear risk |
| LZhou | 2024 | Low risk | Low risk | Unclear risk | Unclear risk | Low risk | Low risk | Unclear risk |

A: Random sequence generation (selection bias); B: Allocation concealment (selection bias); C: Blinding of participants and personnel (performance bias); D: Blinding of outcome assessment (detection bias); E: Incomplete outcome data (attrition bias); F: Selective reporting (reporting bias); G: Other bias

Table S3 Grade results

| Outcome | Number of studies | Study design | Inconsistency | Indirectness | Imprecision | Other considerations | Certainty |
| --- | --- | --- | --- | --- | --- | --- | --- |
| PUQE (Pregnancy-Unique Quantification of Emesis and Nausea) Score | 12 | Randomized controlled study | not serious | not serious | not serious | serious | Low |
| Efficacy | 28 | Randomized controlled study | not serious | not serious | not serious | serious | Moderate |
| Nausea and Vomiting of Pregnancy Quality of Life Scale | 10 | Randomized controlled study | not serious | not serious | not serious | serious | Low |

Table S4 PUQE League Table

| MD 95%CI | | | | | | | | | |
| --- | --- | --- | --- | --- | --- | --- | --- | --- | --- |
| AA |  |  |  |  |  |  |  |  |  |
| 0.15 (-0.45, 0.75) | Acupressure |  |  |  |  |  |  |  |  |
| -0.22 (-0.87, 0.43) | -0.37 (-1.13, 0.39) | ATAA |  |  |  |  |  |  |  |
| 0.24 (-0.57, 1.06) | 0.09 (-0.81, 1) | 0.46 (-0.48, 1.4) | GMAA |  |  |  |  |  |  |
| -0.25 (-1.04, 0.54) | -0.4 (-1.28, 0.49) | -0.03 (-0.95, 0.89) | -0.49 (-1.53, 0.55) | MOX_AA |  |  |  |  |  |
| -1.24 (-1.67, -0.81)* | -1.39 (-1.97, -0.81)* | -1.02 (-1.65, -0.39)* | -1.48 (-2.29, -0.68)* | -0.99 (-1.77, -0.21)* | PN |  |  |  |  |
| -0.08 (-1.11, 0.95) | -0.23 (-1.34, 0.87) | 0.14 (-0.99, 1.28) | -0.32 (-1.55, 0.91) | 0.17 (-1.06, 1.39) | 1.16 (0.14, 2.18)* | SAAA |  |  |  |
| -0.12 (-0.96, 0.72) | -0.27 (-1.19, 0.67) | 0.1 (-0.86, 1.06) | -0.36 (-1.44, 0.72) | 0.13 (-0.93, 1.19) | 1.12 (0.29, 1.95)* | -0.04 (-1.29, 1.21) | TCM_PN |  |  |
| 0.54 (-0.27, 1.36) | 0.39 (-0.51, 1.3) | 0.76 (-0.17, 1.7) | 0.3 (-0.76, 1.37) | 0.79 (-0.25, 1.84) | 1.78 (0.98, 2.59)* | 0.62 (-0.61, 1.85) | 0.66 (-0.42, 1.74) | TFM |  |
| -2.01 (-2.33, -1.69)* | -2.16 (-2.67, -1.65) | -1.79 (-2.35, -1.23)* | -2.25 (-3, -1.5)* | -1.76 (-2.48, -1.04)* | -0.77 (-1.06, -0.48)* | -1.93 (-2.91, -0.95)* | -1.89 (-2.66, -1.12)* | -2.55 (-3.3, -1.8)* | UT |

* Means P<0.05; AA: Acupoint Application; PN: Press needle; WA: Warm acupuncture; Mox: Moxibustion; TCM: Traditional Chinese medicine; TFM: Thunder fire Moxibustion; GMAA: Ginger moxibustion Acupoint Application; ATAA: Auriculotherapy Acupoint Application; SAAA: Scalp Acupuncture Acupoint Application; UT: Usual care.

Table S5 Efficacy League Table

| OR 95%CI | | | | | | | | | | |
| --- | --- | --- | --- | --- | --- | --- | --- | --- | --- | --- |
| AA |  |  |  |  |  |  |  |  |  |  |
| 0.22 (0.03, 0.93)* | AA_WA |  |  |  |  |  |  |  |  |  |
| 0.44 (0.19, 0.97)* | 1.97 (0.42, 15.88) | Acupuncture |  |  |  |  |  |  |  |  |
| 1.09 (0.2, 4.74) | 5 (0.58, 55.03) | 2.51 (0.42, 12.14) | Acupuncture_mox |  |  |  |  |  |  |  |
| 0.33 (0.01, 2.78) | 1.5 (0.04, 26.91) | 0.75 (0.03, 6.85) | 0.29 (0.01, 4.46) | GMAA |  |  |  |  |  |  |
| 0.84 (0.26, 2.48) | 3.81 (0.65, 34.66) | 1.92 (0.6, 5.95) | 0.77 (0.13, 5.27) | 2.56 (0.24, 83.69) | Mox |  |  |  |  |  |
| 0.66 (0.02, 5.87) | 3.01 (0.09, 55.46) | 1.52 (0.05, 14.44) | 0.6 (0.02, 9.14) | 2.01 (0.04, 98.05) | 0.78 (0.02, 8.76) | Mox_AA |  |  |  |  |
| 0.96 (0.36, 2.4) | 4.39 (0.86, 36.26) | 2.21 (0.74, 6.46) | 0.88 (0.17, 5.59) | 2.94 (0.31, 90.75) | 1.15 (0.31, 4.52) | 1.46 (0.14, 46.05) | PN |  |  |  |
| 0.43 (0.11, 1.35) | 1.96 (0.3, 18.42) | 0.99 (0.23, 3.55) | 0.39 (0.06, 2.77) | 1.32 (0.11, 42.88) | 0.51 (0.1, 2.35) | 0.65 (0.05, 21.42) | 0.45 (0.1, 1.76) | TCM_acupuncture |  |  |
| 0.66 (0.02, 5.85) | 2.99 (0.09, 56.47) | 1.51 (0.05, 14.59) | 0.6 (0.02, 9.1) | 2.01 (0.04, 107.46) | 0.78 (0.03, 8.71) | 1 (0.02, 52.32) | 0.68 (0.02, 6.95) | 1.52 (0.05, 18.2) | TFM |  |
| 3.76 (2.45, 5.95)* | 17 (4.38, 124.47)* | 8.65 (4.48, 17.79) * | 3.44 (0.85, 18.18) | 11.45 (1.44, 328.96) * | 4.5 (1.68, 13.63) * | 5.68 (0.68, 165.75) | 3.91 (1.77, 9.45) * | 8.7 (3.1, 32.03) * | 5.71 (0.67, 153.19) | UT |

* Means P<0.05; AA: Acupoint Application; PN: Press needle; WA: Warm acupuncture; Mox: Moxibustion; TCM: Traditional Chinese medicine; TFM: Thunder fire Moxibustion; GMAA: Ginger moxibustion Acupoint Application; ATAA: Auriculotherapy Acupoint Application; SAAA: Scalp Acupuncture Acupoint Application; UT: Usual care.

Table S6 NVPQOl League Table

| MD 95%CI | | | | | | | | |
| --- | --- | --- | --- | --- | --- | --- | --- | --- |
| AA |  |  |  |  |  |  |  |  |
| -4.29 (-9.44, 0.9) | AA_WA |  |  |  |  |  |  |  |
| 0.87 (-7.15, 8.89) | 5.15 (-1.82, 12.14) | ATAA |  |  |  |  |  |  |
| 0.1 (-8.07, 8.27) | 4.38 (-2.74, 11.5) | -0.76 (-10.18, 8.64) | GMAA |  |  |  |  |  |
| 5.12 (-1.93, 12.15) | 9.39 (3.6, 15.16) * | 4.22 (-4.19, 12.72) | 5.02 (-3.54, 13.56) | Mox_AA |  |  |  |  |
| -7.07 (-14.96, 0.82) | -2.8 (-9.6, 3.98) | -7.95 (-17.11, 1.2) | -7.18 (-16.47, 2.14) | -12.2 (-20.48, -3.83) | PN |  |  |  |
| 30.43 (24.2, 36.65)* | 34.71 (29.97, 39.48) * | 29.56 (21.8, 37.33) * | 30.34 (22.44, 38.22) * | 25.32 (18.56, 32.06) * | 37.52 (29.89, 45.08) * | TCM_acupuncture |  |  |
| -6.48 (-12.58, -0.41) * | -2.21 (-6.81, 2.43) | -7.35 (-15.08, 0.35) | -6.58 (-14.42, 1.26) | -11.6 (-18.23, -4.92) | 0.59 (-6.99, 8.14) | -36.92 (-42.67, -31.14) * | TCM_PN |  |
| -12.11 (-16.73, -7.49) * | -7.83 (-10.14, -5.5) * | -12.98 (-19.57, -6.37) * | -12.2 (-18.93, -5.46) * | -17.22 (-22.49, -11.9) * | -5.04 (-11.42, 1.37) | -42.54 (-46.7, -38.38) * | -5.61 (-9.62, -1.63) * | UT |

* Means P<0.05; AA: Acupoint Application; PN: Press needle; WA: Warm acupuncture; Mox: Moxibustion; TCM: Traditional Chinese medicine; TFM: Thunder fire Moxibustion; GMAA: Ginger moxibustion Acupoint Application; ATAA: Auriculotherapy Acupoint Application; SAAA: Scalp Acupuncture Acupoint Application; UT: Usual care.


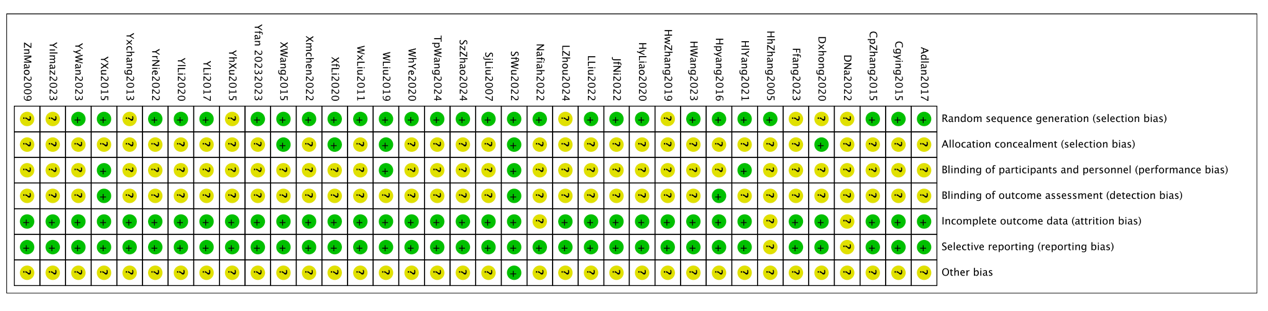


Figure S1 risk of bias summary


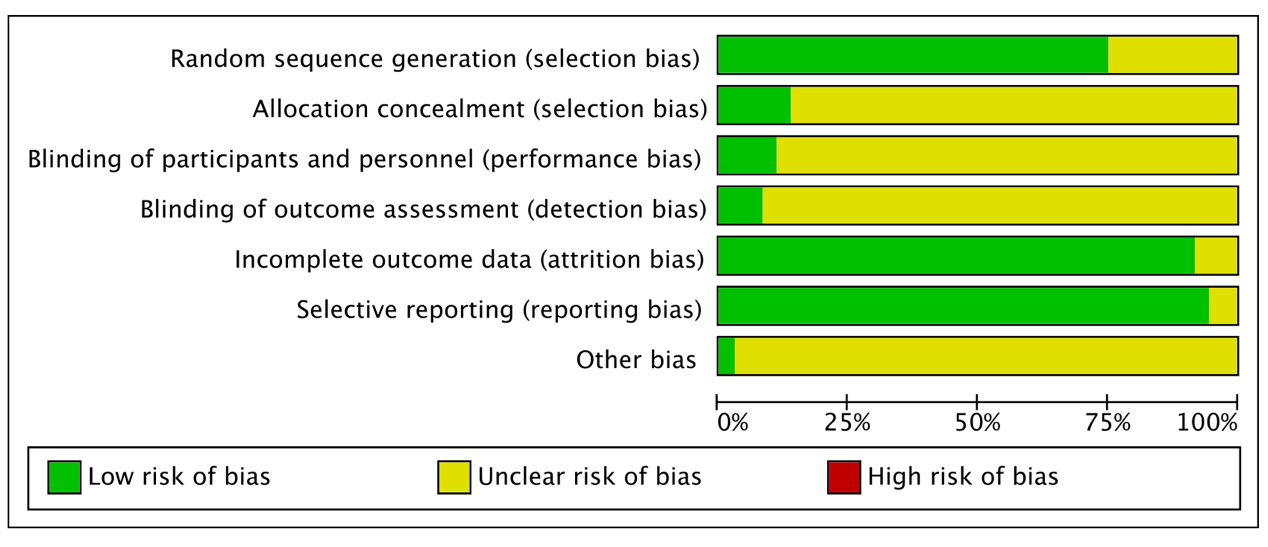


Figure S2 risk of bias graph


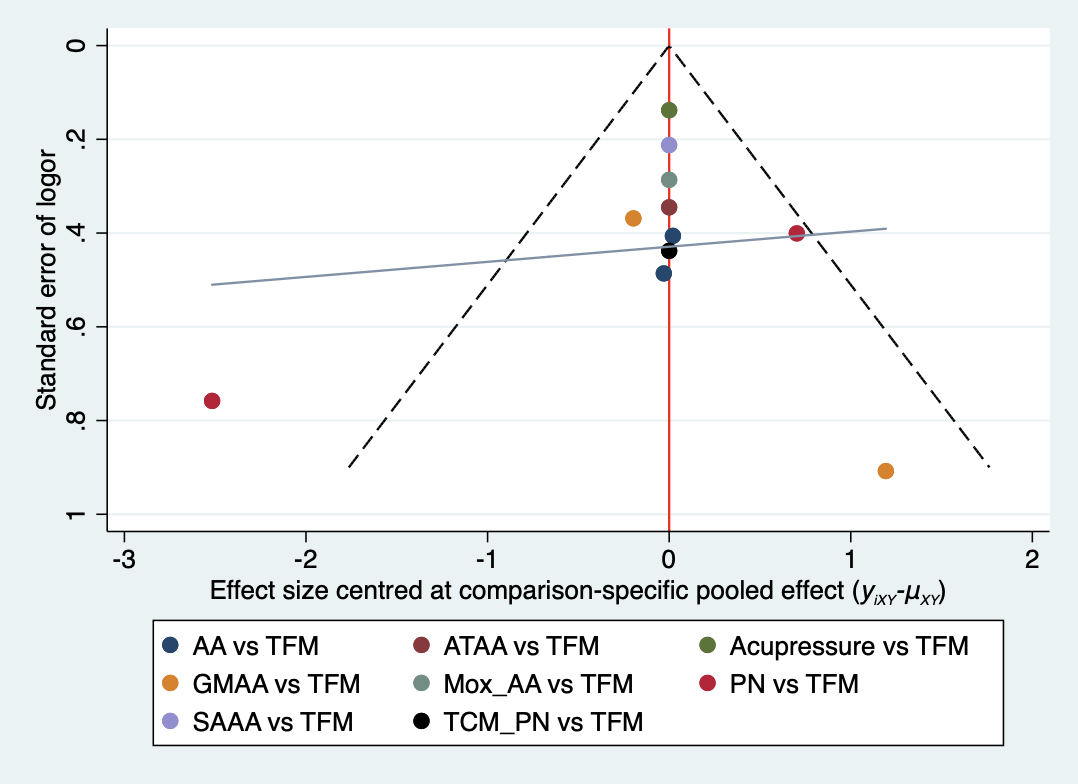


figure S3 PUQE scores funnel plot


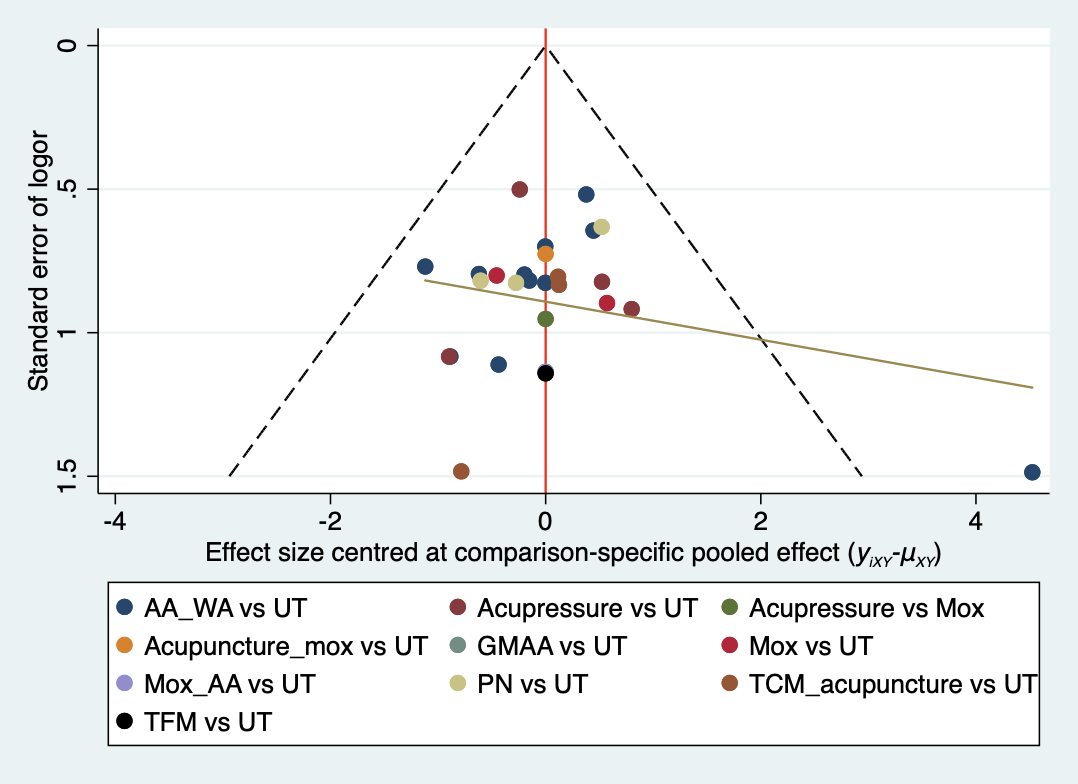


Figure S4 efficacy funnel plot


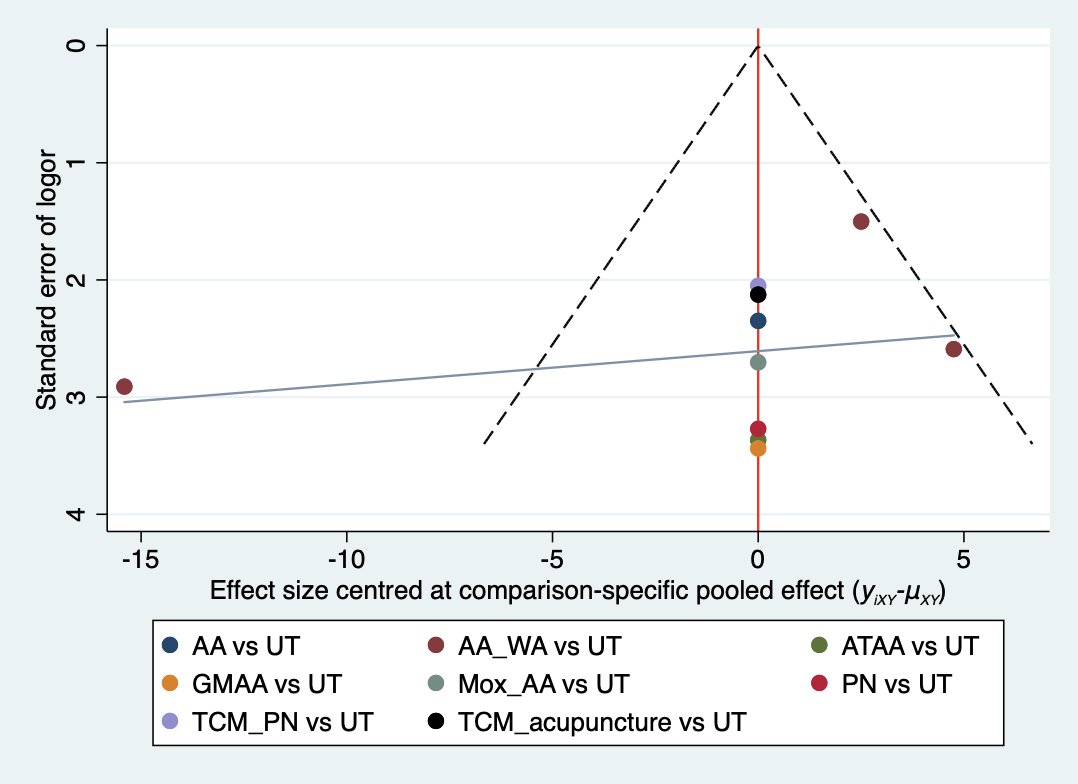


Figure S5 Nausea and Vomiting of Pregnancy Quality of Life funnel plot
